# Supplementary figures and images for: Staphylococcus aureus α-Toxin Induces Acid Sphingomyelinase Release From a Human Endothelial Cell Line
Source: Front Microbiol. 2021 Jul 29;12:694489. doi: 10.3389/fmicb.2021.694489 (PMC8358437; doi:10.3389/fmicb.2021.694489)

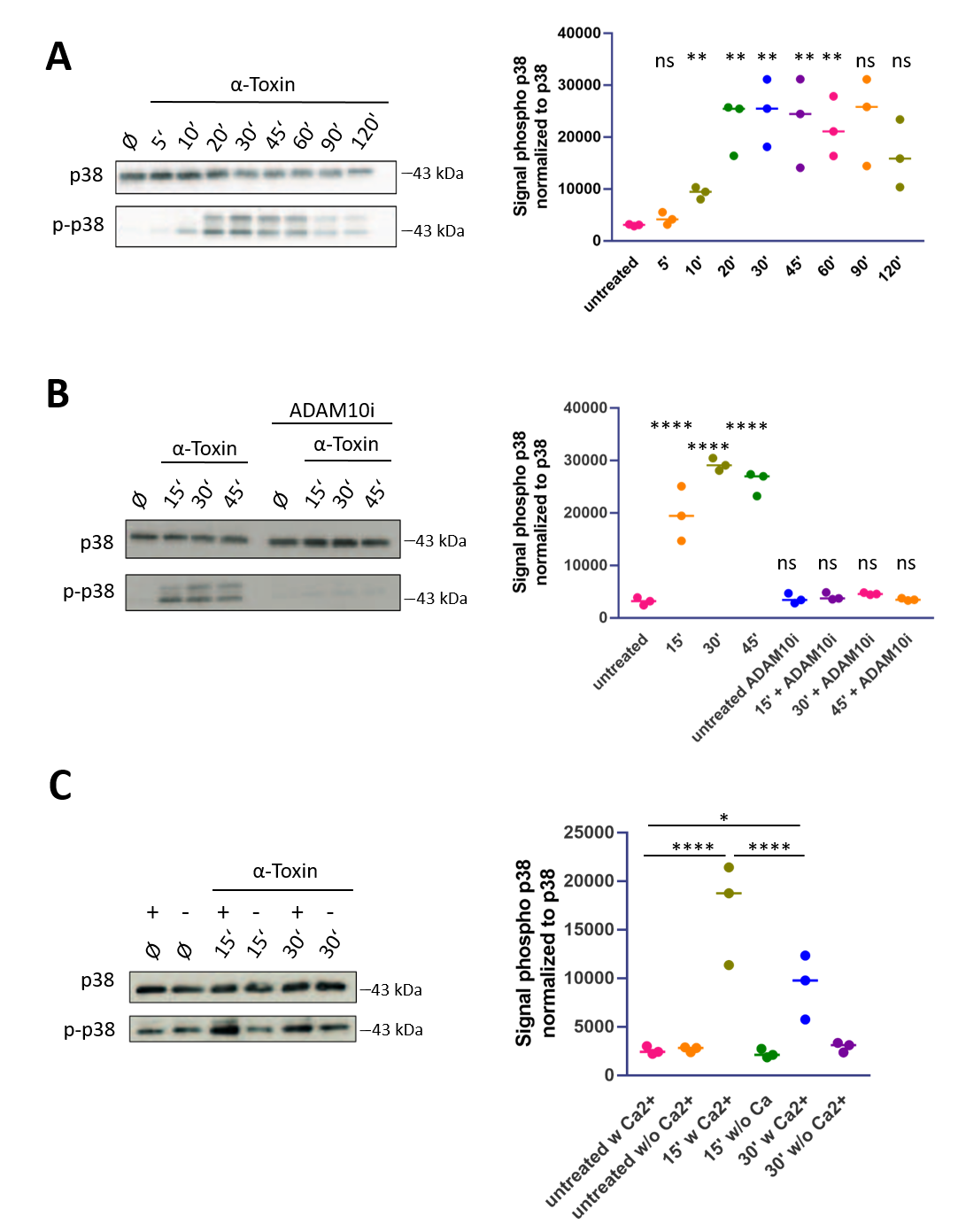

Supplement: Supplementary Figure 1 — α-toxin induces p38 MAPK phosphorylation which is dependent on divalent cations in the medium and ADMA10: bEnd.3 were incubated with 10 μg/ml α-toxin for 5–120 min and phosphorylation of p38 was determined by Western blot (A–C). Pre-treatment of cells with the ADAM10 inhibitor GI254023X inhibited p38 phosphorylation (B). Ca2+ is required for p38 activation, since a buffer lacking Ca2+ reduced α-toxin induced p38 phosphorylation. Shown are representative Western blots of three independent experiments. Phosphorylation of p38 was determined densitometrically and was normalized by signals measured for total p38. p38 is activated significantly during a period ranging from 10 to 60 min post-intoxication (A). (B) Pre-treatment of cells with the ADAM10 inhibitor GI254023X inhibited p38 phosphorylation significantly at all investigated time points. (C) Ca2+ is required for p38 activation, which is observed at 15 and 30 min after intoxication. Activation is not observed in buffers lacking Ca2+. Statistical analysis was performed with GraphPad Prism using One way ANOVA followed by Sidka’ multiple comparison test. ∗: p < 0.05; ∗∗: p < 0.001; ****: p < 0.0001. [file Image_1.TIF]

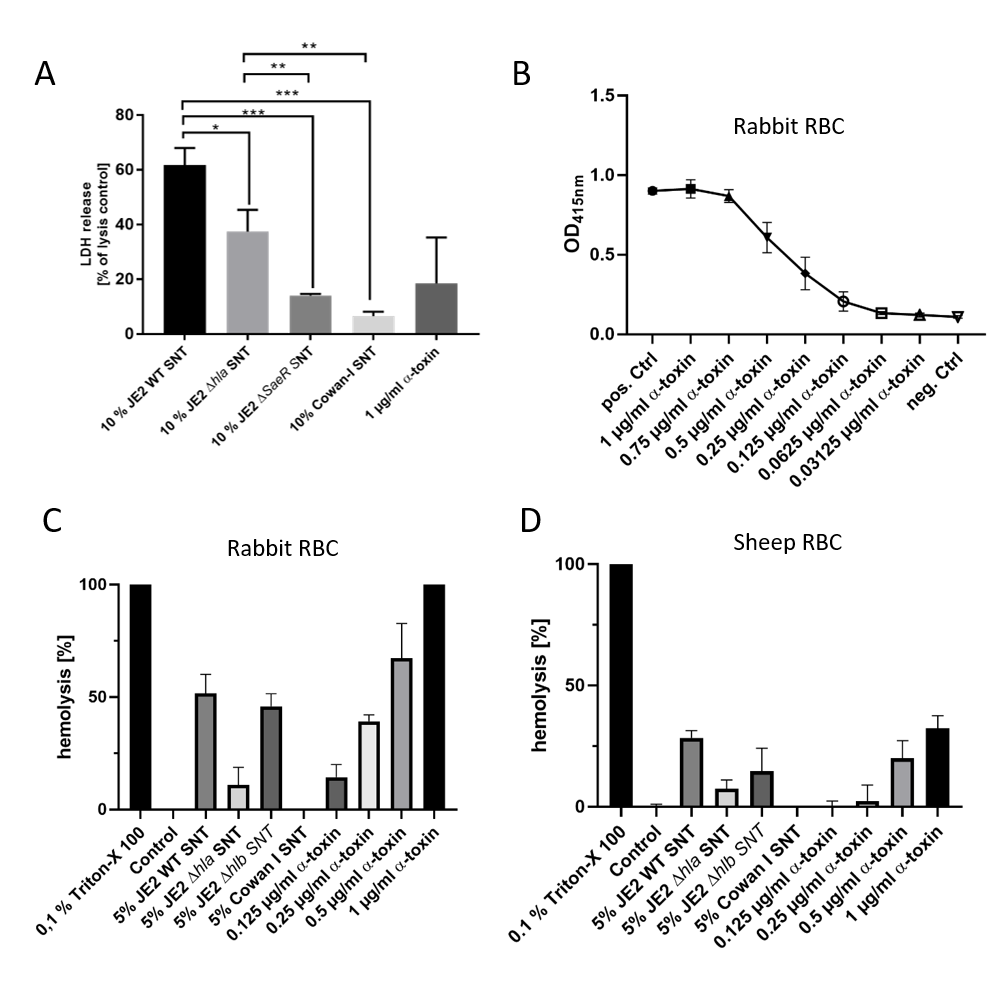

Supplement: Supplementary Figure 2 — THP-1 monocyte cytotoxicity and hemolysis of bacterial culture supernatants and α-toxin. (A) Cytotoxicity of S. aureus JE2 SNT is reduced when compared to SNT of its isogenic α-toxin mutant (Δhla), Cowan I, or a saeR mutant (ΔsaeR), whereas 1 μg/ml α-toxin lysed host cells. Statistical analysis was performed with GraphPad Prism using One-way ANOVA and a post-hoc unpaired Student’s t-tests. ∗: p < 0.05; ∗∗: p < 0.001; ∗∗∗: p < 0.0001. Hemolysis assays were performed using washed rabbit (B,C) and sheep blood (D). For details see main manuscript text. [file Image_2.TIF]

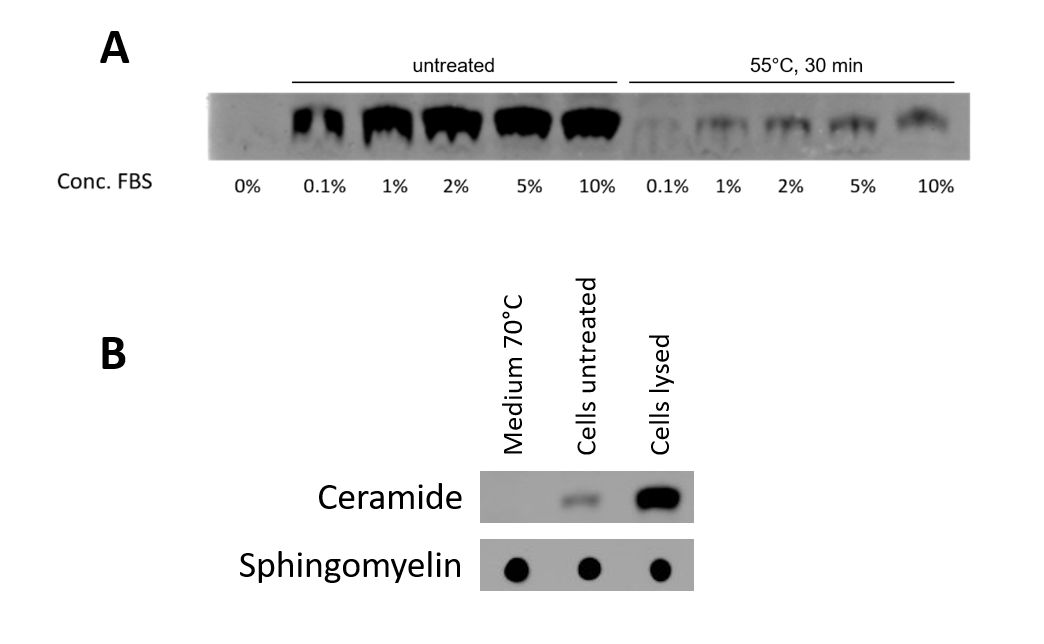

Supplement: Supplementary Figure 3 — FBS exhibits a strong ASM activity which is inactivated upon treatment at elevated temperatures. (A) MCDB131 medium supplemented with untreated of heat inactivated (55°C for 30 min) FBS was tested for its ASM activity. Heat-inactivation for 30 min at 55°C reduced ASM activity. (B) Heat inactivation for 1 h at 70°C (Medium 70°C) abolishes ASM activity of FBS completely. Thin-layer results are representative images of multiple experiments. [file Image_3.TIF]

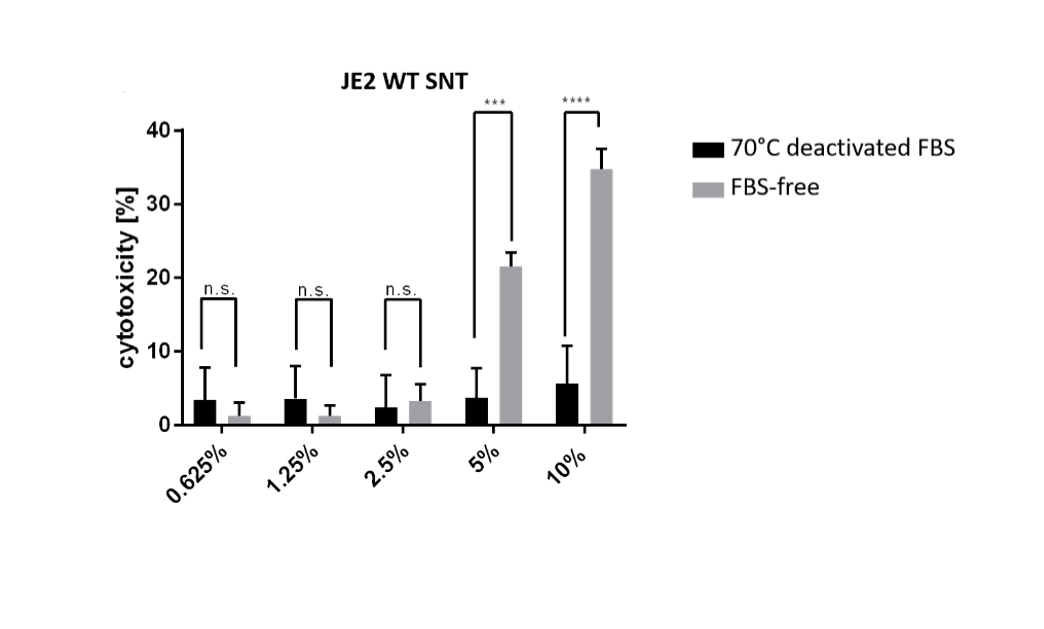

Supplement: Supplementary Figure 4 — S. aureus phenol soluble modulins are inhibited by heat-treated FBS. Epithelial cells were challenged with increasing concentrations of culture supernatants of S. aureus overnight cultures diluted in cell culture media. Additionally 70°C-treated FBS was added or the assays were left FBS-free. The heat-treated FBS shows the same inhibition of cytotoxicity as normal FBS. n = 3. Significance was calculated with one-way ANOVA followed by post-hoc Student’s t-tests. ∗∗∗: p < 0.001; ns: not significant. [file Image_4.TIF]

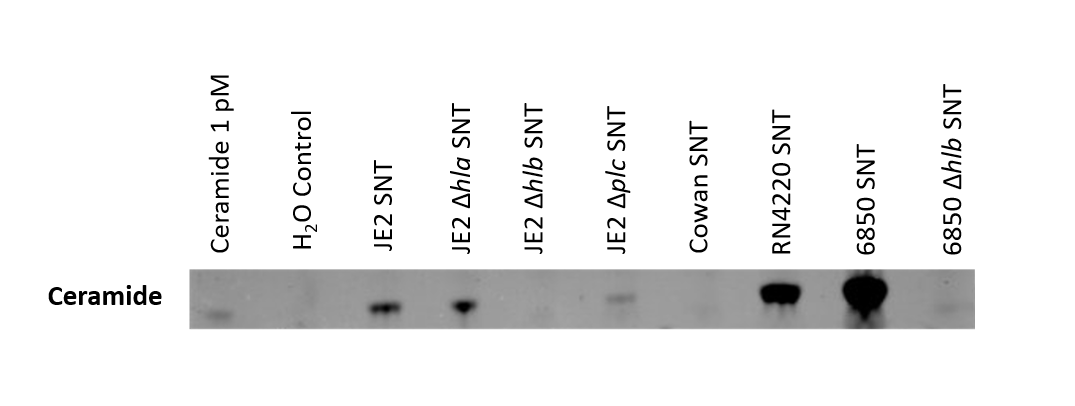

Supplement: Supplementary Figure 5 — S. aureus exhibits toxin specific sphingomyelinase activity. Equal amounts of supernatants (SNT) from S. aureus overnight cultures were tested for sphingomyelinase activity. S. aureus exhibited a strain-specific conversion of sphingomyelin to ceramide with strains 6850 and RN4220 showing strongest bSMase activities. Even the clinical strain JE2 demonstrates measurable activities, although the responsible ORF hlb is inactivated by phage lysogeny in this strain suggesting phage reactivation. Sphingomyelinase activity is absent from insertional mutants within hlb (termed “Δhlb”) in strain JE2 and 6850, as well as in strain Cowan I. Thin-layer results are representative images of three independent experiments. [file Image_5.TIF]

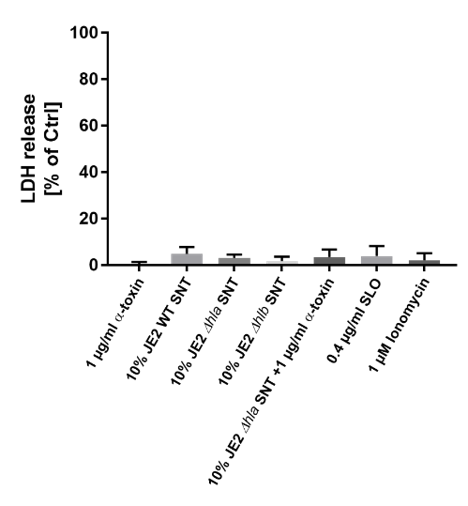

Supplement: Supplementary Figure 6 — Lactate dehydrogenase is not released from HuLEC treated with bacterial supernatants. HuLEC were either challenged with 10% bacterial culture supernatant of S. aureus JE2 WT (SNT WT), JE2 lacking α-toxin (Δhla SNT) or JE2 lacking β-toxin (Δhlb SNT), were treated with 1 μg/ml of purified α-toxin, or were left untreated (Ctrl). Cell supernatants were collected and were tested for LDH release. Shown is LDH release as per cent of the lysis control. LDH assays of the culture supernatants demonstrate that the endothelial cells remained intact over the course of the experiment. [file Image_6.TIF]

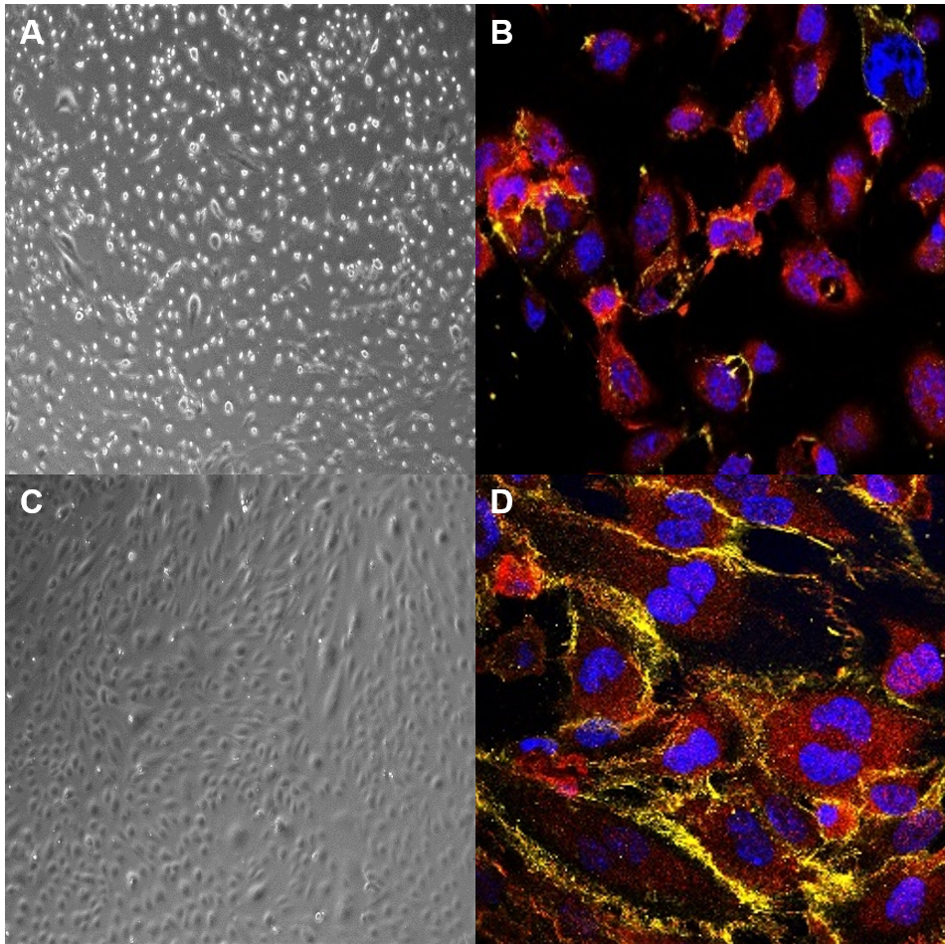

Supplement: Supplementary Figure 7 — S. aureus JE2 Δhla supernatant does not induce tight junction degradation and endothelial cell death. HuLEC were seeded 7 days prior to toxin challenge on glass cover slips coated with collagen. Cells were either treated with 10% bacterial culture supernatants of wild-type S. aureus JE2 (A,B) or its isogenic Δhla mutant (C,D). Phase contrast microscopy images (A,C) were taken at 10× magnification. Immunofluorescence images (B,D) taken at 40× magnification. Blue – Hoechst 33258; Red – ZO-1; Yellow – VE-Cadherin. [file Image_7.TIF]

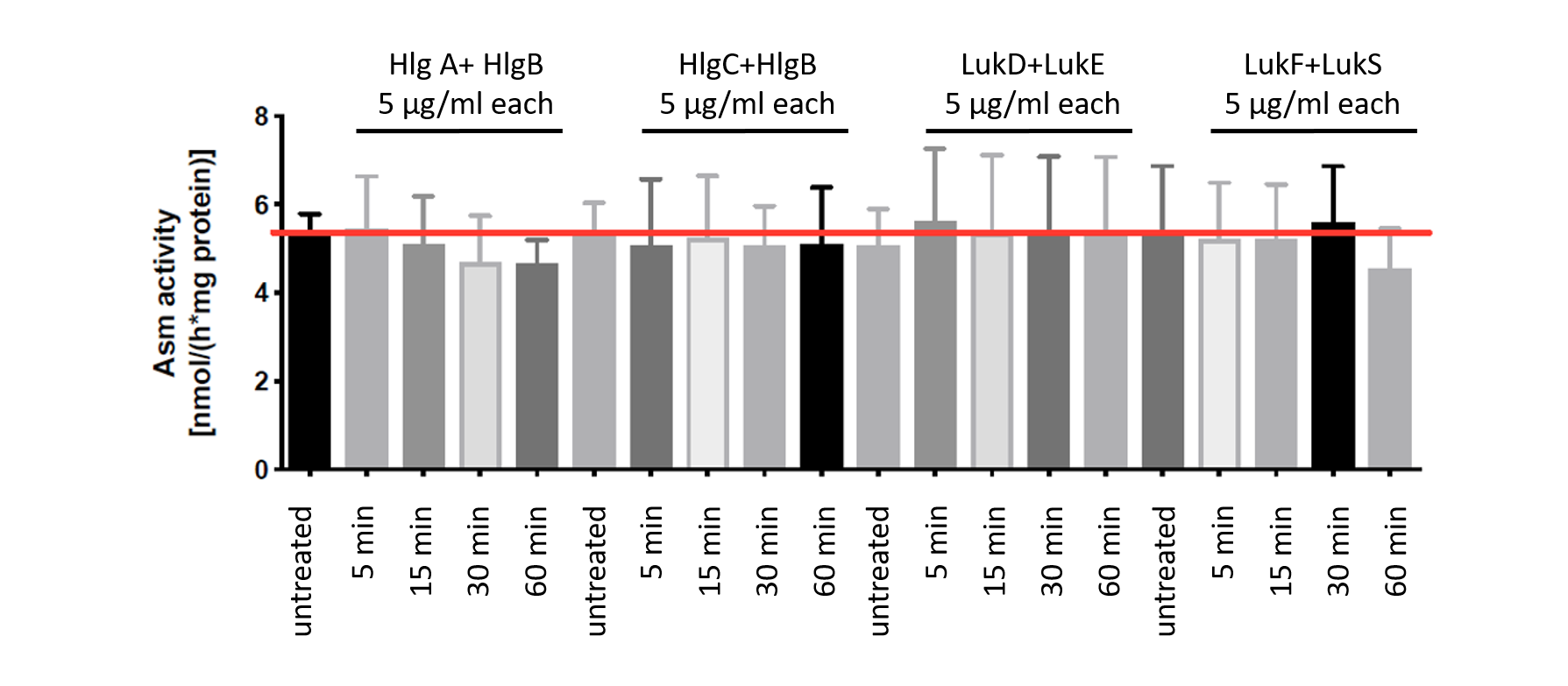

Supplement: Supplementary Figure 8 — Purified S. aureus toxins other than α-toxin fail to induce ASM activation in murine endothelial cells. bEnd.3 cells were challenged with various combinations of hemolysin γ (Hlg) or leukocidin (Luk) subunits for 5–60 min and ASM activity was measured in cell lysate. n = 3. [file Image_8.TIF]
